# Author Correction: CHD3 helicase domain mutations cause a neurodevelopmental syndrome with macrocephaly and impaired speech and language

**DOI:** 10.1038/s41467-019-10161-9

**Published:** 2019-05-02

**Authors:** Lot Snijders Blok, Justine Rousseau, Joanna Twist, Sophie Ehresmann, Motoki Takaku, Hanka Venselaar, Lance H. Rodan, Catherine B. Nowak, Jessica Douglas, Kathryn J. Swoboda, Marcie A. Steeves, Inderneel Sahai, Connie T. R. M. Stumpel, Alexander P. A. Stegmann, Patricia Wheeler, Marcia Willing, Elise Fiala, Aaina Kochhar, William T. Gibson, Ana S. A. Cohen, Ruky Agbahovbe, A. Micheil Innes, P. Y. Billie Au, Julia Rankin, Ilse J. Anderson, Steven A. Skinner, Raymond J. Louie, Hannah E. Warren, Alexandra Afenjar, Boris Keren, Caroline Nava, Julien Buratti, Arnaud Isapof, Diana Rodriguez, Raymond Lewandowski, Jennifer Propst, Ton van Essen, Murim Choi, Sangmoon Lee, Jong H. Chae, Susan Price, Rhonda E. Schnur, Ganka Douglas, Ingrid M. Wentzensen, Christiane Zweier, André Reis, Martin G. Bialer, Christine Moore, Marije Koopmans, Eva H. Brilstra, Glen R. Monroe, Koen L. I. van Gassen, Ellen van Binsbergen, Ruth Newbury-Ecob, Lucy Bownass, Ingrid Bader, Johannes A. Mayr, Saskia B. Wortmann, Kathy J. Jakielski, Edythe A. Strand, Katja Kloth, Tatjana Bierhals, The DDD study, Jeremy F. McRae, Stephen Clayton, Tomas W. Fitzgerald, Joanna Kaplanis, Elena Prigmore, Diana Rajan, Alejandro Sifrim, Stuart Aitken, Nadia Akawi, Mohsan Alvi, Kirsty Ambridge, Daniel M. Barrett, Tanya Bayzetinova, Philip Jones, Wendy D. Jones, Daniel King, Netravathi Krishnappa, Laura E. Mason, Tarjinder Singh, Adrian R. Tivey, Munaza Ahmed, Uruj Anjum, Hayley Archer, Ruth Armstrong, Jana Awada, Meena Balasubramanian, Siddharth Banka, Diana Baralle, Angela Barnicoat, Paul Batstone, David Baty, Chris Bennett, Jonathan Berg, Birgitta Bernhard, A. Paul Bevan, Maria Bitner-Glindzicz, Edward Blair, Moira Blyth, David Bohanna, Louise Bourdon, David Bourn, Lisa Bradley, Angela Brady, Simon Brent, Carole Brewer, Kate Brunstrom, David J. Bunyan, John Burn, Natalie Canham, Bruce Castle, Kate Chandler, Elena Chatzimichali, Deirdre Cilliers, Angus Clarke, Susan Clasper, Jill Clayton-Smith, Virginia Clowes, Andrea Coates, Trevor Cole, Irina Colgiu, Amanda Collins, Morag N. Collinson, Fiona Connell, Nicola Cooper, Helen Cox, Lara Cresswell, Gareth Cross, Yanick Crow, Mariella D’Alessandro, Tabib Dabir, Rosemarie Davidson, Sally Davies, Dylan de Vries, John Dean, Charu Deshpande, Gemma Devlin, Abhijit Dixit, Angus Dobbie, Alan Donaldson, Dian Donnai, Deirdre Donnelly, Carina Donnelly, Angela Douglas, Sofia Douzgou, Alexis Duncan, Jacqueline Eason, Sian Ellard, Ian Ellis, Frances Elmslie, Karenza Evans, Sarah Everest, Tina Fendick, Richard Fisher, Frances Flinter, Nicola Foulds, Andrew Fry, Alan Fryer, Carol Gardiner, Lorraine Gaunt, Neeti Ghali, Richard Gibbons, Harinder Gill, Judith Goodship, David Goudie, Emma Gray, Andrew Green, Philip Greene, Lynn Greenhalgh, Susan Gribble, Rachel Harrison, Lucy Harrison, Victoria Harrison, Rose Hawkins, Liu He, Stephen Hellens, Alex Henderson, Sarah Hewitt, Lucy Hildyard, Emma Hobson, Simon Holden, Muriel Holder, Susan Holder, Georgina Hollingsworth, Tessa Homfray, Mervyn Humphreys, Jane Hurst, Ben Hutton, Stuart Ingram, Melita Irving, Lily Islam, Andrew Jackson, Joanna Jarvis, Lucy Jenkins, Diana Johnson, Elizabeth Jones, Dragana Josifova, Shelagh Joss, Beckie Kaemba, Sandra Kazembe, Rosemary Kelsell, Bronwyn Kerr, Helen Kingston, Usha Kini, Esther Kinning, Gail Kirby, Claire Kirk, Emma Kivuva, Alison Kraus, Dhavendra Kumar, V. K. Ajith Kumar, Katherine Lachlan, Wayne Lam, Anne Lampe, Caroline Langman, Melissa Lees, Derek Lim, Cheryl Longman, Gordon Lowther, Sally A. Lynch, Alex Magee, Eddy Maher, Alison Male, Sahar Mansour, Karen Marks, Katherine Martin, Una Maye, Emma McCann, Vivienne McConnell, Meriel McEntagart, Ruth McGowan, Kirsten McKay, Shane McKee, Dominic J. McMullan, Susan McNerlan, Catherine McWilliam, Sarju Mehta, Kay Metcalfe, Anna Middleton, Zosia Miedzybrodzka, Emma Miles, Shehla Mohammed, Tara Montgomery, David Moore, Sian Morgan, Jenny Morton, Hood Mugalaasi, Victoria Murday, Helen Murphy, Swati Naik, Andrea Nemeth, Louise Nevitt, Andrew Norman, Rosie O’Shea, Caroline Ogilvie, Kai-Ren Ong, Soo-Mi Park, Michael J. Parker, Chirag Patel, Joan Paterson, Stewart Payne, Daniel Perrett, Julie Phipps, Daniela T. Pilz, Martin Pollard, Caroline Pottinger, Joanna Poulton, Norman Pratt, Katrina Prescott, Abigail Pridham, Annie Procter, Hellen Purnell, Oliver Quarrell, Nicola Ragge, Raheleh Rahbari, Josh Randall, Lucy Raymond, Debbie Rice, Leema Robert, Eileen Roberts, Jonathan Roberts, Paul Roberts, Gillian Roberts, Alison Ross, Elisabeth Rosser, Anand Saggar, Shalaka Samant, Julian Sampson, Richard Sandford, Ajoy Sarkar, Susann Schweiger, Richard Scott, Ingrid Scurr, Ann Selby, Anneke Seller, Cheryl Sequeira, Nora Shannon, Saba Sharif, Charles Shaw-Smith, Emma Shearing, Debbie Shears, Eamonn Sheridan, Ingrid Simonic, Roldan Singzon, Zara Skitt, Audrey Smith, Kath Smith, Sarah Smithson, Linda Sneddon, Miranda Splitt, Miranda Squires, Fiona Stewart, Helen Stewart, Volker Straub, Mohnish Suri, Vivienne Sutton, Ganesh Jawahar Swaminathan, Elizabeth Sweeney, Kate Tatton-Brown, Cat Taylor, Rohan Taylor, Mark Tein, I. Karen Temple, Jenny Thomson, Marc Tischkowitz, Susan Tomkins, Audrey Torokwa, Becky Treacy, Claire Turner, Peter Turnpenny, Carolyn Tysoe, Anthony Vandersteen, Vinod Varghese, Pradeep Vasudevan, Parthiban Vijayarangakannan, Julie Vogt, Emma Wakeling, Sarah Wallwark, Jonathon Waters, Astrid Weber, Diana Wellesley, Margo Whiteford, Sara Widaa, Sarah Wilcox, Emily Wilkinson, Denise Williams, Nicola Williams, Louise Wilson, Geoff Woods, Christopher Wragg, Michael Wright, Laura Yates, Michael Yau, Chris Nellåker, Michael Parker, Helen V. Firth, Caroline F. Wright, David R. FitzPatrick, Jeffrey C. Barrett, Matthew E. Hurles, John D. Roberts, Robert M. Petrovich, Shinichi Machida, Hitoshi Kurumizaka, Stefan Lelieveld, Rolph Pfundt, Sandra Jansen, Pelagia Deriziotis, Laurence Faivre, Julien Thevenon, Mirna Assoum, Lawrence Shriberg, Tjitske Kleefstra, Han G. Brunner, Paul A. Wade, Simon E. Fisher, Philippe M. Campeau

**Affiliations:** 1 Department of Human Genetics, Radboud University Medical Center, Nijmegen, 6500HB The Netherlands; 2 Language and Genetics Department, Max Planck Institute for Psycholinguistics, Nijmegen, 6500AH The Netherlands; 3 Donders Institute for Brain, Cognition and Behaviour, Radboud University, Nijmegen, 6500HE The Netherlands; 4 CHU Sainte-Justine Research Center, Montreal, QC H3T 1C5 Canada; 5 National Institute of Environmental Health Sciences, Research Triangle Park, NC 27709 USA; 6 Centre for Molecular and Biomolecular Informatics, Radboud Institute for Molecular Life Sciences, Radboud University Medical Center, Nijmegen, 6500HB The Netherlands; 7 Division of Genetics and Genomics, Boston Children’s Hospital, Boston, MA 02115 USA; 8 Department of Neurology, Massachusetts General Hospital and Harvard Medical School, Boston, MA 02114 USA; 9 Department of Medical Genetics, Massachusetts General Hospital, Boston, MA 02114 USA; 10 Department of Clinical Genetics and GROW-School for Oncology and Developmental Biology, Maastricht University Medical Center, Maastricht, 6202AZ The Netherlands; 11 Nemours Childrens Clinic, Orlando, FL 32827 USA; 12 Division of Genetics and Genomic Medicine, Department of Pediatrics, Washington University School of Medicine, St. Louis, MO 63110 USA; 13 Valley Children’s Hospital, Madera, CA 93636 USA; 14 British Columbia Children’s Hospital Research Institute, Vancouver, BC V5Z 4H4 Canada; 15 Department of Medical Genetics, University of British Columbia, Vancouver, BC V6H 3N1 Canada; 16 Department of Medical Genetics and Alberta Children’s Hospital Research Institute, Cumming School of Medicine, University of Calgary, Calgary, AB T2N 4N1 Canada; 17 Department of Clinical Genetics, Royal Devon and Exeter NHS Foundation Trust (Heavitree), Exeter, EX2 5DW UK; 18 Division of Genetics, Department of Medicine, University of Tennessee Medical Center, Knoxville, TN 37920 USA; 19 Greenwood Genetic Center, Greenwood, SC 29646 USA; 20 GRC ConCer-LD, Sorbonne Universités, UPMC Univ Paris ; Department of Medical Genetics and Centre de Référence Malformations et maladies congénitales du cervelet et déficiences intellectuelles de causes rares, Armand Trousseau Hospital, GHUEP, AP-HP, Paris, 75012 France; 21 AP-HP, Hôpital de la Pitié-Salpêtrière, Département de Génétique, Paris, 75013 France; 22 Groupe de Recherche Clinique (GRC) ‘déficience intellectuelle et autisme’ UPMC, Paris, 75005 France; 23 INSERM, U 1127, CNRS UMR 7225, Institut du Cerveau et de la Moelle épinière, ICM, Sorbonne Universités, UPMC Univ Paris 06 UMR S 1127, 75013 Paris, France; 24 GRC ConCer-LD, Sorbonne Universités, UPMC Univ Paris 06; Department Child Neurology and Reference Center for Neuromuscular Diseases “Nord/Est/Ile-de-France”, FILNEMUS, Armand Trousseau Hospital, GHUEP, AP-HP, Paris, 75012 France; 25 GRC ConCer-LD, Sorbonne Universités, UPMC Univ Paris 06; Department of Child Neurology and National Reference Center for Neurogenetic Disorders, Armand Trousseau Hospital, GHUEP, AP-HP, INSERM U1141, 75012 Paris, France; 26 Clinical Genetics Division, Virginia Commonwealth University Health System, Richmond, VA 23298 USA; 27 Clinical Genetics Department, University Medical Center Groningen, Groningen, 9700RB The Netherlands; 28 Department of Biomedical Sciences, Seoul National University College of Medicine, Seoul, 08826 Republic of Korea; 29 Department of Pediatrics, Seoul National University College of Medicine, Seoul National University Children’s Hospital, Seoul, 08826 Republic of Korea; 30 Oxford University Hospitals NHS Foundation Trust, Oxford, OX3 7HE UK; 31 GeneDx, Gaithersburg, MD 20877 USA; 32 Institute of Human Genetics, Friedrich-Alexander-Universität Erlangen-Nürnberg, Erlangen, 91054 Germany; 33 Northwell Health, Division of Medical Genetics and Genomics, Great Neck, NY 11021 USA; 34 Department of Genetics, University Medical Center Utrecht, Utrecht University, Utrecht, 3508AB The Netherlands; 35 University Hospitals Bristol, Department of Clinical Genetics, St Michael’s Hospital, Bristol, BS2 8EG UK; 36 Department of Clinical Genetics, University Children’s Hospital, Paracelsus Medical University, Salzburg, A-5020 Austria; 37 Department of Pediatrics, Salzburger Landeskliniken and Paracelsus Medical University, Salzburg, A-5020 Austria; 38 Institute of Human Genetics, Technische Universität München, Munich, 81675 Germany; 39 Institute of Human Genetics, Helmholtz Zentrum München, Neuherberg, 85764 Germany; 40 Communication Sciences and Disorders, Augustana College, Rock Island, IL 61201 USA; 41 Department of Neurology, Mayo Clinic, Rochester, MN 55905 USA; 42 Institute of Human Genetics, University Medical Center Hamburg-Eppendorf, Hamburg, 20246 Germany; 43 Waseda University, Tokyo, 169-8050 Japan; 44 Equipe Génétique des Anomalies du Développement, Université de Bourgogne-Franche Comté, Dijon, 21070 France; 45 Centre de Génétique et Centre de Référence Anomalies du Développement et Syndromes Malformatifs, FHU TRANSLAD, Hôpital d’Enfants, CHU Dijon et Université de Bourgogne, Dijon, 21079 France; 46 Waisman Center, Phonology Project, Madison, WI 53705-2280 USA; 47 Sainte-Justine Hospital, University of Montreal, Montreal, QC H3T 1C5 Canada; 48 Wellcome Trust Sanger Institute, Wellcome Trust Genome Campus, Hinxton, Cambridge, CB10 1SA UK; 49 MRC Human Genetics Unit, MRC IGMM, University of Edinburgh, Western General Hospital, Edinburgh, EH4 2XU UK; 50 Department of Engineering Science, University of Oxford, Parks Road, Oxford, OX1 3PJ UK; 51 Wessex Clinical Genetics Service, University Hospital Southampton, Princess Anne Hospital, Coxford Road, Southampton, SO16 5YA UK; 52 Wessex Regional Genetics Laboratory, Salisbury NHS Foundation Trust, Salisbury District Hospital, Odstock Road, Salisbury, Wiltshire SP2 8BJ UK; 53 Faculty of Medicine, University of Southampton, Building 85, Life Sciences Building, Highfield Campus, Southampton, SO17 1BJ UK; 54 South West Thames Regional Genetics Centre, St George’s Healthcare NHS Trust, St George’s, University of London, Cranmer Terrace, London, SW17 0RE UK; 55 Institute of Medical Genetics, University Hospital of Wales, Heath Park, Cardiff, CF14 4XW UK; 56 Department of Clinical Genetics, Block 12, Glan Clwyd Hospital, Rhyl, Denbighshire LL18 5UJ UK; 57 East Anglian Medical Genetics Service, Box 134, Cambridge University Hospitals NHS Foundation Trust, Cambridge Biomedical Campus, Cambridge, CB2 0QQ UK; 58 Sheffield Regional Genetics Services, Sheffield Children’s NHS Trust, Western Bank, Sheffield, S10 2TH UK; 59 Manchester Centre for Genomic Medicine, St Mary’s Hospital, Central Manchester University Hospitals NHSFoundation Trust, Manchester Academic Health Science Centre, Manchester, M13 9WL UK; 60 North East Thames Regional Genetics Service, Great Ormond Street Hospital for Children NHS Foundation Trust, Great Ormond Street Hospital, Great Ormond Street, London, WC1N3JH UK; 61 North of Scotland Regional Genetics Service, NHS Grampian, Department of Medical Genetics Medical School, Foresterhill, Aberdeen, AB25 2ZD UK; 62 East of Scotland Regional Genetics Service, Human Genetics Unit, Pathology Department, NHS Tayside, Ninewells Hospital, Dundee, DD1 9SY UK; 63 Yorkshire Regional Genetics Service, Leeds Teaching Hospitals NHS Trust, Department of Clinical Genetics, Chapel Allerton Hospital, Chapeltown Road, Leeds, LS7 4SA UK; 64 North West Thames Regional Genetics Centre, North West London Hospitals NHS Trust, The Kennedy Galton Centre, Northwick Park and St Mark’s NHS Trust Watford Road, Harrow, HA1 3UJ UK; 65 Oxford Regional Genetics Service, Oxford Radcliffe Hospitals NHS Trust, The Churchill Old Road, Oxford, OX3 7LJ UK; 66 West Midlands Regional Genetics Service, Birmingham Women’s NHS Foundation Trust, Birmingham Women’s Hospital, Edgbaston, Birmingham B15 2TG UK; 67 Northern Genetics Service, Newcastle upon Tyne Hospitals NHS Foundation Trust, Institute of Human Genetics, International Centre for Life, Central Parkway, Newcastle upon Tyne, NE1 3BZ UK; 68 Northern Ireland Regional Genetics Centre, Belfast Health and Social Care Trust, Belfast City Hospital, Lisburn Road, Belfast, BT9 7AB UK; 69 Peninsula Clinical Genetics Service, Royal Devon and Exeter NHS Foundation Trust, Clinical Genetics Department, Royal Devon & Exeter Hospital (Heavitree), Gladstone Road, Exeter, EX1 2ED UK; 70 South East Thames Regional Genetics Centre, Guy’s and St Thomas’ NHS Foundation Trust, Guy’s Hospital, Great Maze Pond, London, SE1 9RT UK; 71 Leicestershire Genetics Centre, University Hospitals of Leicester NHS Trust, Leicester Royal Infirmary (NHS Trust), Leicester, LE1 5WW UK; 72 Nottingham Regional Genetics Service, City Hospital Campus, Nottingham University Hospitals NHS Trust, The Gables, Hucknall Road, Nottingham, NG5 1PB UK; 73 West of Scotland Regional Genetics Service, NHS Greater Glasgow and Clyde, Institute of Medical Genetics, Yorkhill Hospital, Glasgow, G3 8SJ UK; 74 Bristol Genetics Service (Avon, Somerset, Gloucs and West Wilts), University Hospitals Bristol NHS Foundation Trust, St Michael’s Hospital, St Michael’s Hill, Bristol, BS2 8DT UK; 75 Merseyside and Cheshire Genetics Service, Liverpool Women’s NHS Foundation Trust, Department of Clinical Genetics, Royal Liverpool Children’s Hospital Alder Hey, Eaton Road, Liverpool, L12 2AP UK; 76 National Centre for Medical Genetics, Our Lady’s Children’s Hospital, Crumlin, Dublin 12, Ireland; 77 Department of Clinical Genetics, Block 12, Glan Clwyd Hospital, Rhyl, Denbighshire, LL18 5UJ Wales UK; 78 Nuffield Department of Obstetrics & Gynaecology, University of Oxford, Level 3, Women’s Centre, John Radcliffe Hospital, Oxford, OX3 9DU UK; 79 Institute of Biomedical Engineering, Department of Engineering Science, University of Oxford, Old Road Campus Research Building, Oxford, OX3 7DQ UK; 80 Big Data Institute, University of Oxford, Roosevelt drive, Oxford, OX3 7LF UK; 81 The Ethox Centre, Nuffield Department of Population Health, University of Oxford, Old Road Campus, Oxford, OX3 7LF UK

**Keywords:** Clinical epigenetics, Disease genetics, Neurodevelopmental disorders, Chromatin remodelling

Correction to: *Nature Communications* 10.1038/s41467-018-06014-6, published online 05 November 2018.

The HTML and PDF versions of this Article were updated after publication to remove images of one individual from Fig. 1.

