# Supplementary material for: CHD3 helicase domain mutations cause a neurodevelopmental syndrome with macrocephaly and impaired speech and language
Source: Nat Commun. 2018 Nov 5;9:4619. doi: 10.1038/s41467-018-06014-6 (PMC6218476; doi:10.1038/s41467-018-06014-6)
Supplement: Supplementary file 1 — Supplementary Information [file 41467_2018_6014_MOESM1_ESM.pdf]

Supplementary Information

CHD3 helicase domain mutations cause a neurodevelopmental syndrome with macrocephaly and impaired speech and language

Snijders Blok et al.

Supplementary Figure 1: Conservation of mutated amino acids and clustering around conserved SNF2-motifs

```
sp|Q12873|CHD3_HUMAN  TQPRFITATGGTLHMYQLEGLNWLRFSWAQGTDTILADEMGLGKTIQTIVFLYSLYKEGH 783
sp|Q14839|CHD4_HUMAN  RQPEYLDATGGTLHPYQMEGLNWLRFSWAQGTDTILADEMGLGKTVQTAVFLYSLYKEGH 773
sp|O16102|CHD3_DROME  DQPVFLKEAGLKLHPFQIEGVSWLRYSWGQGIPTILADEMGLGKTIQTVVFLYSLFKEGH 314
sp|Q22516|CHD3_CAEEL  VQPDFISETGGNLHPYQLEGINWLRHCWSNGTDAILADEMGLGKTVQSLTFLYTLMKEGH 663
sp|P22082|SNF2_YEAST  KQPS--ILVGGTLKDYQIKGLQWMVSLFNNHLNGILADEMGLGKTIQTISLLTYLYEMKN 814
                       **     .* .*: :*::*:.*:   : :    ***********:*   :*  * :  :
                                                              I

sp|Q12873|CHD3_HUMAN  TKGPFLVSAPLSTIINWEREFQMWAPKFYVVTYTGDKDSRAIIRENEFSFEDNAIKGGKK 843
sp|Q14839|CHD4_HUMAN  SKGPFLVSAPLSTIINWEREFEMWAPDMYVVTYVGDKDSRAIIRENEFSFEDNAIRGGKK 833
sp|O16102|CHD3_DROME  CRGPFLISVPLSTLTNWERELELWAPELYCVTYVGGKTARAVIRKHEISFEEVTTKTM-- 372
sp|Q22516|CHD3_CAEEL  TKGPFLIAAPLSTIINWEREAELWCPDFYVVTYVGDRESRMVIREHEFSFVDGAVRGGPK 723
sp|P22082|SNF2_YEAST  IRGPYLVIVPLSTLSNWSSEFAKWAPTLRTISFKGSPNERKAK----------------- 857
                       :**:*: .****: **. *   *.* :  ::: *.   *
                               Ia

sp|Q12873|CHD3_HUMAN  AFKMKREAQVKFHVLLTSYELITIDQAALGSIRWACLVVDEAHRLKNNQSKFFRVL-NGY 902
sp|Q14839|CHD4_HUMAN  ASRMKKEASVKFHVLLTSYELITIDMAILGSIDWACLIVDEAHRLKNNQSKFFRVL-NGY 892
sp|O16102|CHD3_DROME  ---RENQTQYKFNVMLTSYEFISVDAAFLGCIDWAALVVDEAHRLRSNQSKFFRIL-SKY 428
sp|Q22516|CHD3_CAEEL  VSKIKTLENLKFHVLLTSYECINMDKAILSSIDWAALVVDEAHRLKNNQSTFFKNL-REY 782
sp|P22082|SNF2_YEAST  ---QAKIRAGEFDVVLTTFEYIIKERALLSKVKWVHMIIDEGHRMKNAQSKLSLTLNTHY 914
                                :*.*:**::* *  : * *. : *. :::**.**::. **.:   *   *
                                                            II

sp|Q12873|CHD3_HUMAN  KIDHKLLLTGTPLQNNLEELFHLLNFLTPERFNNLEGFLEEFADI--------------- 947
sp|Q14839|CHD4_HUMAN  SLQHKLLLTGTPLQNNLEELFHLLNFLTPERFHNLEGFLEEFADI--------------- 937
sp|O16102|CHD3_DROME  RIGYKLLLTGTPLQNNLEELFHLLNFLSSGKFNDLQTFQAEFTDV--------------- 473
sp|Q22516|CHD3_CAEEL  NIQYRVLLTGTPLQNNLEELFHLLNFLAPDRFNQLESFTAEFSEI--------------- 827
sp|P22082|SNF2_YEAST  HADYRLILTGTPLQNNLPELWALLNFVLPKIFNSVKSFDEWFNTPFANTGGQDKIELSEE 974
                         ::::********** **: ****:    *:.:: *   *
                                 III

sp|Q12873|CHD3_HUMAN  SKEDQIKKLHDLLGPHMLRRLKADVFKNMPAKTELIVRVELSPMQKKYYKYILTRNFEAL 1007
sp|Q14839|CHD4_HUMAN  AKEDQIKKLHDMLGPHMLRRLKADVFKNMPSKTELIVRVELSPMQKKYYKYILTRNFEAL 997
sp|O16102|CHD3_DROME  SKEEQVKRLHEILEPHMLRRLKADVLKSMPPKSEFIVRVELSSMQKKFYKHILTKNFKAL 533
sp|Q22516|CHD3_CAEEL  SKEDQIEKLHNLLGPHMLRRLKADVLTGMPSKQELIVRVELSAMQKKYYKNILTRNFDAL 887
sp|P22082|SNF2_YEAST  ETLLVIRRLHKVLRPFLLRRLKKDVEKELPDKVEKVVKCKMSALQQIMYQQMLKYRRLFI 1034
                       .   :.:**.:* *.:***** ** . :* * * :*: ::* :*:  *: :*. .   :
                                         IV

sp|Q12873|CHD3_HUMAN  NSRGGG---NQVSLLNIMMDLKKCCNHPYLFPVAAMESPKLPSGAYEGGALIKSSGKLML 1064
sp|Q14839|CHD4_HUMAN  NARGGG---NQVSLLNVVMDLKKCCNHPYLFPVAAMEAPKMPNGMYDGSALIRASGKLLL 1054
sp|O16102|CHD3_DROME  NQKGGG---RVCSLLNIMMDLRKCCNHPYLFPSAAEEATISPSGLYEMSSLTKASGKLDL 590
sp|Q22516|CHD3_CAEEL  NVKNGG---TQMSLINIIMELKKCCNHPYLFMKACLEAPKLKNGMYEGSALIKNAGKFVL 944
sp|P22082|SNF2_YEAST  GDQNNKKMVGLRGFNNQIMQLKKICNHPFVFEEVEDQIN--P-TRETNDDIWRVAGKFEL 1091
                      . :..       .: * :*:*:* ****::*  .  :           . : : :**: *

sp|Q12873|CHD3_HUMAN  LQKMLRKLKEQGHRVLIFSQMTKMLDLLEDFLDYEGYKYERIDGGITGALRQEAIDRFNA 1124
sp|Q14839|CHD4_HUMAN  LQKMLKNLKEGGHRVLIFSQMTKMLDLLEDFLEHEGYKYERIDGGITGNMRQEAIDRFNA 1114
sp|O16102|CHD3_DROME  LSKMLKQLKADNHRVLLFSQMTKMLNVLEHFLEGEGYQYDRIDGSIKGDLRQKAIDRFND 650
sp|Q22516|CHD3_CAEEL  LQKMLRKLKDGGHRVLIFSQMTMMLDILEDFCDVEGYKYERIDGSITGQQRQDAIDRYNA 1004
sp|P22082|SNF2_YEAST  LDRILPKLKATGHRVLIFFQMTQIMDIMEDFLRYINIKYLRLDGHTKSDERSELLRLFNA 1151
                      *.::* :**  .****:* *** :::::*.*    . :* *:**  ..  *.. :  :*

sp|Q12873|CHD3_HUMAN  PGAQQFCFLLSTRAGGLGINLATADTVIIFDSDWNPHNDIQAFSRAHRIGQANKVMIYRF 1184
sp|Q14839|CHD4_HUMAN  PGAQQFCFLLSTRAGGLGINLATADTVIIYDSDWNPHNDIQAFSRAHRIGQNKKVMIYRF 1174
sp|O16102|CHD3_DROME  PVSEHFVFLLSTRAGGLGINLATADTVIIFDSDWNPHNDVQAFSRAHRMGQKKKVMIYRF 710
sp|Q22516|CHD3_CAEEL  PGAKQFVFLLSTRAGGLGINLATADTVIIYDSDWNPHNDIQAFSRAHRLGQKHKVMIYRF 1064
sp|P22082|SNF2_YEAST  PDSEYLCFILSTRAGGLGLNLQTADTVIIFDTDWNPHQDLQAQDRAHRIGQKNEVRILRL 1211
                      * :: : *:*********:** *******:*:*****:*:** .****:** ::* * *:
                                     V                                  VI

sp|Q12873|CHD3_HUMAN  VTRASVEERITQVAKRKMMLTHLVVRPGLGSKAG-SMSKQELDDILKFGTEELFKDENEG 1243
sp|Q14839|CHD4_HUMAN  VTRASVEERITQVAKKKMMLTHLVVRPGLGSKTG-SMSKQELDDILKFGTEELFKDEATD 1233
sp|O16102|CHD3_DROME  VTHNSVEERIMQVAKHKMMLTHLVVRPGMGGMTT-NFSKDELEDILRFGTEDLFKDGK-- 767
sp|Q22516|CHD3_CAEEL  VTKGSVEERITSVAKKKMLLTHLVVRAGLGAKDGKSMSKTELDDVLRWGTEELFKEEEAP 1124
sp|P22082|SNF2_YEAST  ITTNSVEEVILERAYKKLDIDGKVIQAGKFDNKSTSEEQEALLRSLLDAEEERRKKRESG 1271
                      :*  **** * . * :*: :   *:: *       . .:  *   *  . *:  *.
```

Alignment of amino acids 724-1243 of the CHD3 protein with the Swiss-Prot sequences of human CHD4 (Q14839), CHD3 in drosophila melanogaster (O16102), CHD3 in C. Elegans (Q22516) and SNF2 in yeast (P22082). Missense mutations (affected amino acid residues) in our CHD3 cohort are shown in red, while published mutations in CHD4 are shown in orange[1,2]. The majority of missense mutations affect highly conserved amino acid residues. The missense mutations clearly cluster in or around the known conserved SNF2 motifs (motif I, Ia, II, III, IV, V and VI; in figure depicted by boxes with the respective motif number) of the helicase domain. One missense mutation in CHD3 (p.W1158R) affects the same residue as a previously published mutation in CHD4: p.W1148L[1].

Supplementary Figure 2: RNA analysis of the c.5802_5803insGAAC mutation (p.(Phe1935Glufs*108))

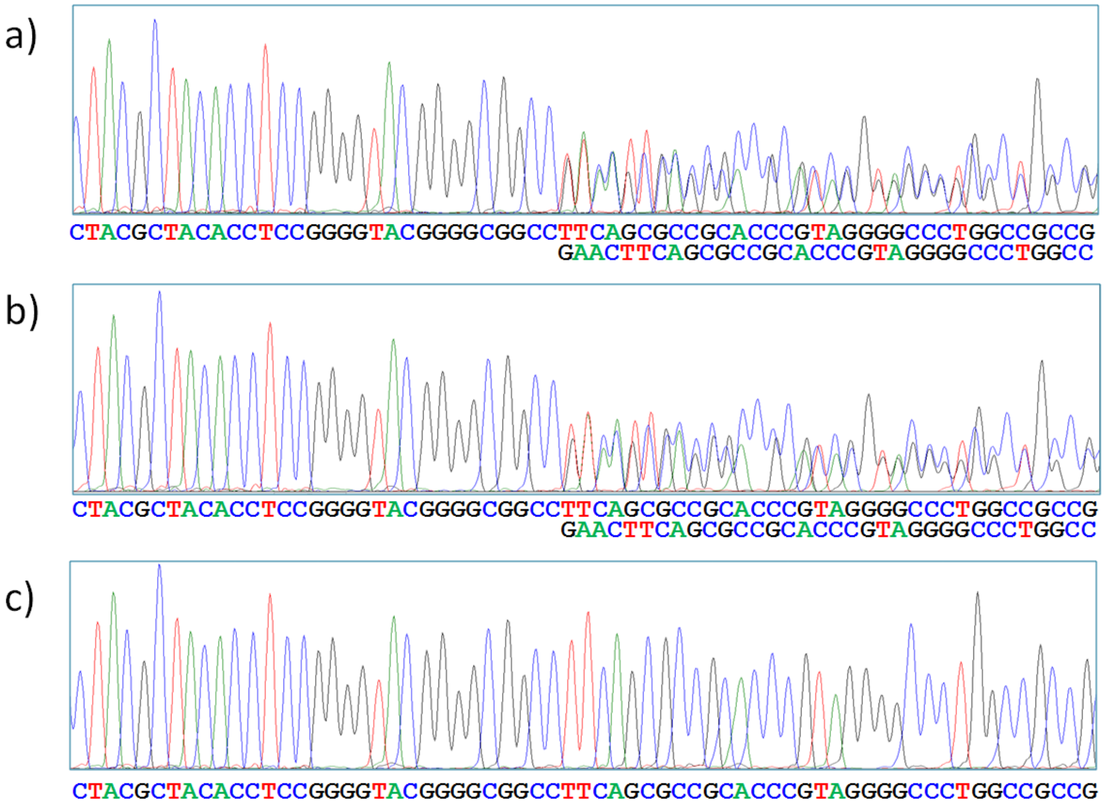

To study the effects of the frameshift mutation in the penultimate exon of the *CHD3* gene [c.5802_5803insGAAC (p.(Phe1935Glufs*108))] in mRNA from individual 35, lymphoblastoid cell lines were generated from peripheral blood cells by Epstein-Barr virus transformation following standard procedures. To check for the occurrence of nonsense mediated decay, mRNA was isolated from cells that were cultured in the presence (a) and absence (b) of cyclohexamide. A negative control was also included (c). CHD3 mRNA was analyzed by the synthesis of cDNA and Sanger sequencing according to standard protocols. Sanger analysis shows no difference between the RNA analysis from untreated cells and the cells that were treated with cyclohexamide. These data indicate that the alternative transcript that is a result of the frameshift mutation is not degraded by nonsense mediated decay. In conclusion, in this individual (individual 35) two transcripts are present: the wild-type transcript and the transcript with the frameshift, that leads to a stop codon after 108 amino acids.

Supplementary Figure 3: Subcellular localization of wildtype and mutant CHD3

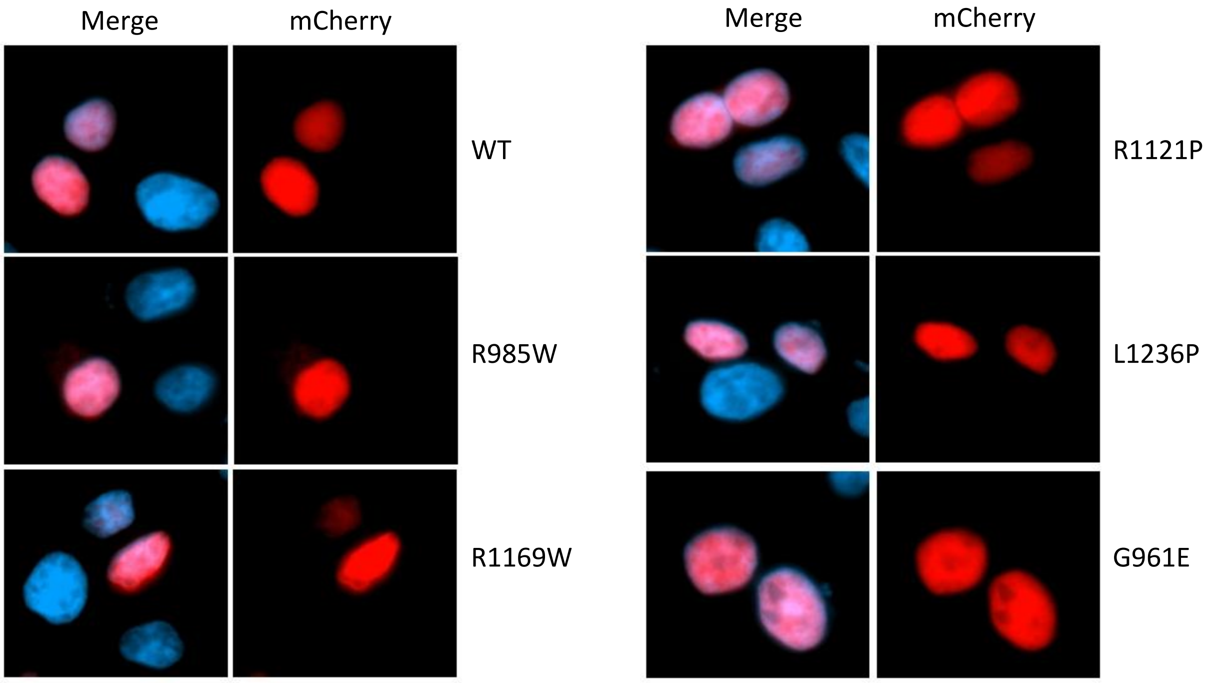

Fluorescence microscopy images of HEK293 cells transfected with wild-type and synthetic CHD3 variants fused to mCherry (shown in red). Nuclei were stained with Hoechst 33342 (blue). The localization of CHD3 is nuclear in all pictures (wild-type and mutations), no difference in subcellular localization was seen between wild-type and mutants.

Supplementary Figure 4: Purification of wildtype and mutant CHD3

a)

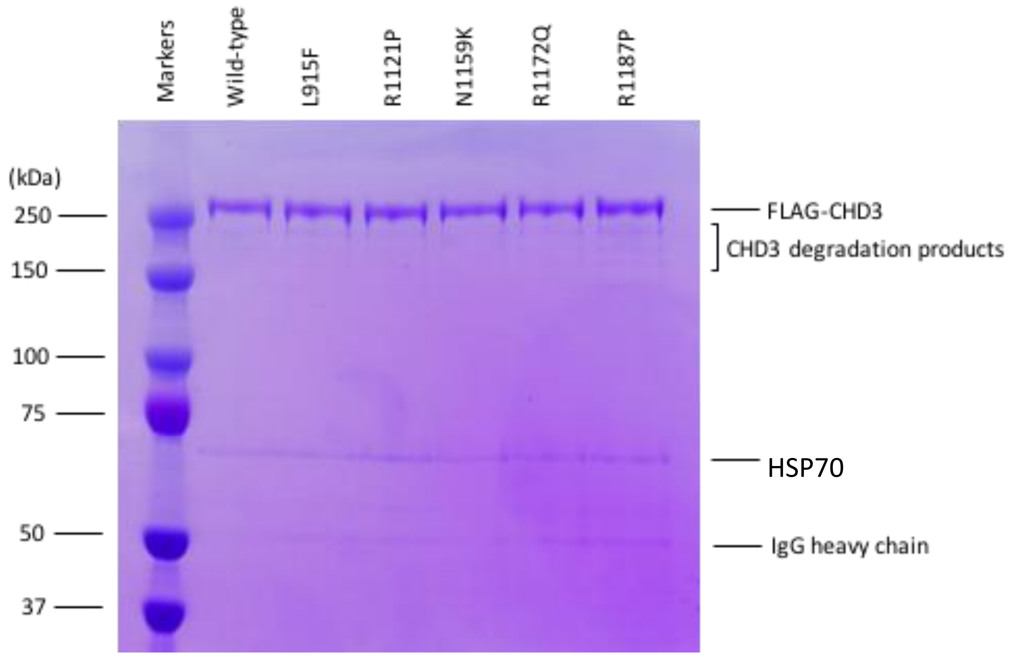

b)

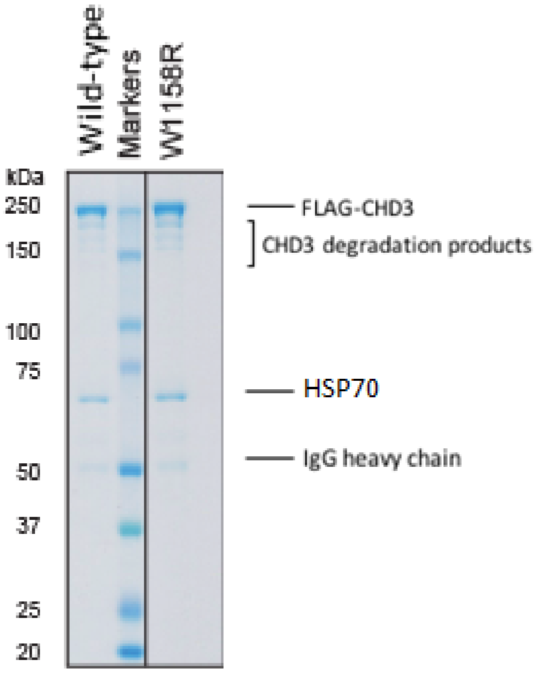

Purified wild-type CHD3 and mutant CHD3. 500 ng of the purified proteins were analyzed by SDS-PAGE and stained with Coomassie Brilliant Blue. Proteins were confirmed by mass spectrometry by the NIEHS Mass Spectrometry Research and Support Group.

a) Lane 1: protein marker; lane 2: wild-type FLAG-CHD3 (~230 kDa); lane 3: p.Leu915Phe; lane 4: p.Arg1121Pro; lane 5: p.Asn1159Lys; lane 6: p.Arg1172Gln; and lane 7: p.Arg1187Pro.

b) Lane 1: wild-type FLAG-CHD3 (~230 kDa); lane 2: protein marker and lane 3: p.Trp1158Arg.

Supplementary Figure 5: ATPase activity in the absence of DNA substrate

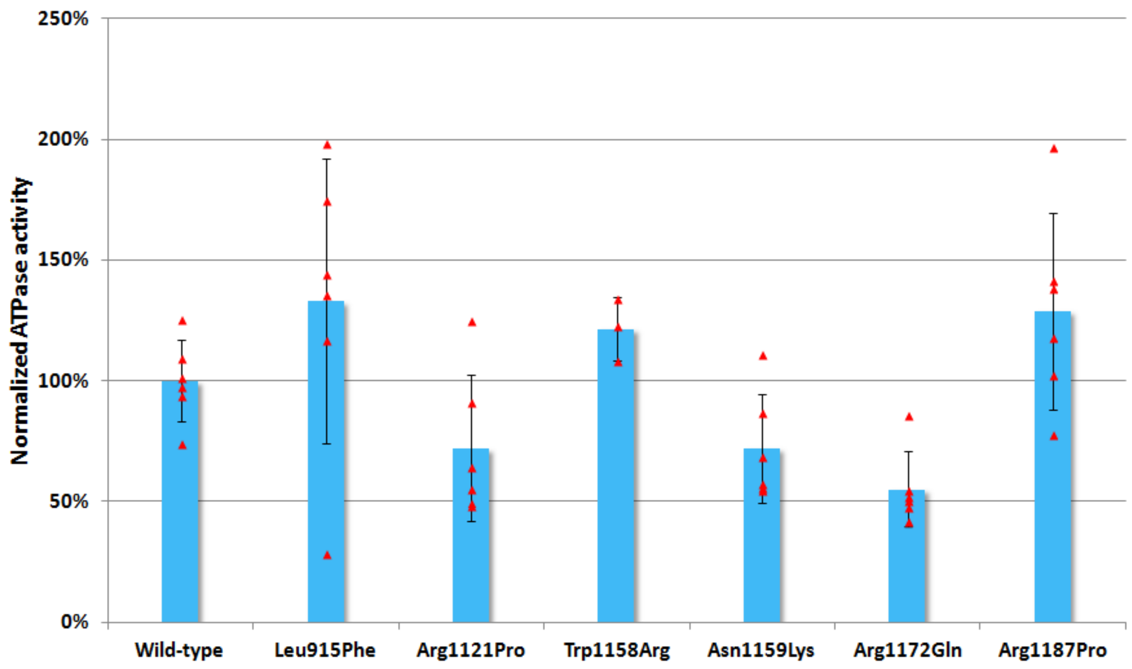

ATPase activities were measured in the absence of DNA substrates. Released phosphate was separated from unhydrolyzed ATP by thin layer chromatography, and detected by exposure to a phosphorimager. The experimental values (percentage hydrolyzed ATP) for the different mutant conditions were normalized to values for the wild-type condition within the experiment, to derive a normalized ATPase activity. Wild-type data points depicted are representative, for individual wild-type replicate values for all experiments please see Supplementary Data 2. All other raw values for these experiments can also be found in Supplementary Data 2. Three independent experiments from two individual purifications (Wild-type, p.Leu915Phe, p.Arg1121Pro, p.Asn1159Lys, p.Arg1172Gln, and p.Arg1187Pro) (N=6) or one purification (p.Trp1158Arg) (N=3) were performed. The experimental data are presented as means +/- standard deviation, individual data points are shown as red triangles.

Supplementary Figure 6: ATPase assay with mixing of wildtype and mutant CHD3

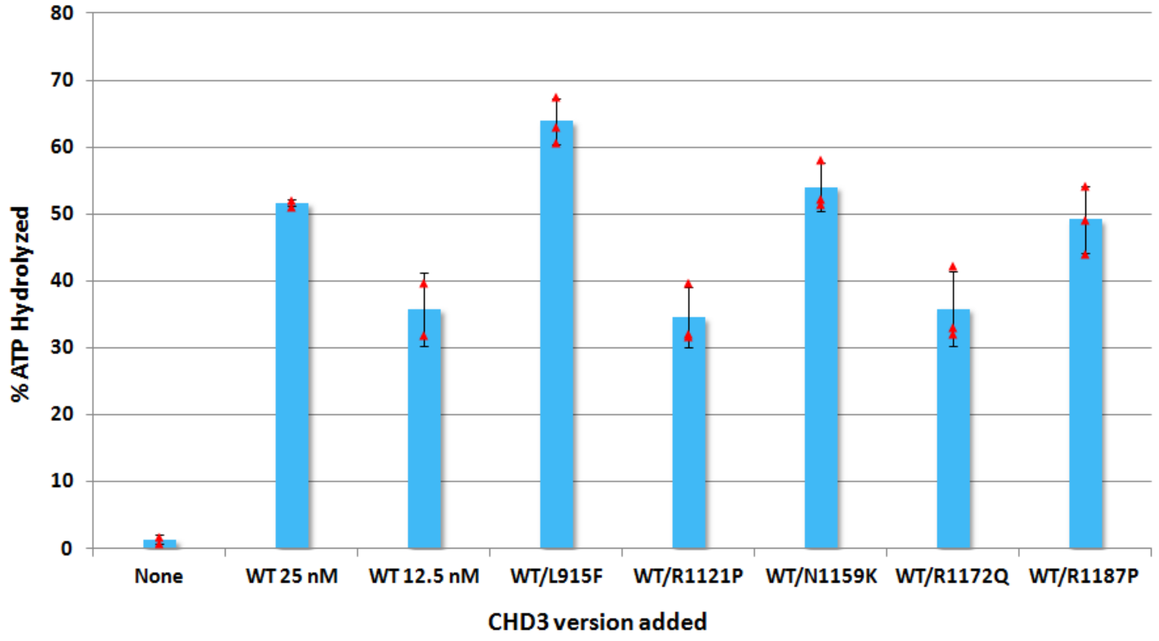

Thin layer chromatography was used to detect nucleosome-dependent ATPase activity. Equimolar concentrations of wild-type (12.5 nM) and mutant protein (12.5 nM) were incubated with nucleosome for 40 minutes. Error bars indicate standard deviations, individual data points are shown as red triangles (N=3 for mixed WT/mutant conditions, N=2 for other conditions). Raw values for these experiments can be found in Supplementary Data 2.

Supplementary Table 1: Primer sequences

Primers used for cloning of FLAG-CHD3 expression constructs		
	Fw (5’ to 3’)	Rv (5’ to 3’)
	gaaaacctgtattttcagggcaaggcggcagacactgtgatcc	gggtccctgaaagaggacttcaaggtcgtctatacagatcacctcccc
Primers used to create CHD3 mutant constructs for ATPase and remodeling assays		
	Fw (5’ to 3’)	Rv (5’ to 3’)
L915F	ctgacaggaaccccatttcagaataatctggagga	tcctccagattattctgaaatggggttcctgtcag
R1121P	ccaggagcattaaacggatcgatggcctcct	aggaggccatcgatccgtttaatgctcctgg
W1158R	aggaacccccataatgacatccagg	gtcagaatcaaagatgatgacagtgtcagc
N1159K	gtcatcatctttgattctgactggaaaccccataatgacat	atgtcattatggggtttccagtcagaatcaaagatgatgac
R1172Q	gccgggctcatcagattggccaggc	gcctggccaatctgatgagcccggc
R1187P	Cttccactgacgcgggagtcacaaaccgg	ccggtttgtgactcccgcgtcagtggaag
Primers used to create CHD3 mutant constructs for immunofluorescence		
	Fw (5’ to 3’)	Rv (5’ to 3’)
R985W	gccaagacagagctcatcgtttgggtggagcta	tagctccacccaaacgatgagctctgtcttggc
R1169W	atccaggcctttagctgggctcatcggattg	caatccgatgagcccagctaaaggcctggat
R1121P	aggaggccatcgatccgtttaatgctcctgg	ccaggagcattaaacggatcgatggcctcct
L1236P	caaatttggcactgaagagccattcaaggatgaaaacgagg	cctcgttttcatccttgaatggctcttcagtgccaaatttg
G961E	tgcatgatttgctggagccacacatgctgcg	cgcagcatgtgtggctccagcaaatcatgca

Supplementary Note 1: Three-dimensional modeling and mutation analysis for de novo CHD3 mutations

Methods

For this analysis, we used the *CHD3 de novo* mutations found in our cohort and the following sequence of interest:

>CHD3_
MKAADTVILWARSKNDQLRISFPPGLCWGDRMPDKDDIRLLPSALGVKKRKRGPKKQKEN
KPGKPRKRKKRDSEEEFGSERDEYREKSESGGSEYGTGPGRKRRRKHREKKEKKTKRRKK
GEGDGGQKQVEQKSSATLLLTWGLEDVEHVFSEEDYHTLTNYKAFSQFMRPLIAKKNPKI
PMSKMMTILGAKWREFSANNPFKGSAAAVAAAAAAAAAVAEQVSAAVSSATPIAPSGPP
ALPPPPAADIQPPPIRRAKTKEGKGPGHKRRSKSPRVPDGRKKLRGKKMAPLKIKLGLLG
GKRKKGGSYVFQSDEGPEPEAEESDLDSGSVHSASGRPDGPVRTKKLKRGRPGRKKKKVL
GCPAVAGEEEVDGYETDHQDYCEVCQQGGEIILCDTCPRAYHLVCLDPELDRAPEGKWSC
PHCEKEGVQWEAKEEEEEYEEEGEEEGEKEEEDDHMEYCRVCKDGGELLCCDACISSYHI
HCLNPPLPDIPNGEWLCPRCTCPVLKGRVQKILHWRWGEPPVAVPAPQQADGNPDVPPPR
PLQGRSEREFFVKWVGLSYWHCSWAKELQLEIFHLVMYRNYQRKNDMDEPPPLDYGSGED
DGKSDKRKVKDPHYAEMEEKYYRFGIKPEWMTVHRIINHSVDKKGNYHYLVKWRDLPYDQ
STWEEDEMNIPEYEEHKQSYWRHRELIMGEDPAQPRKYKKKKKELQGDGPPSSPTNDPTV
KYETQPRFITATGGTLHMYQLEGLNWLRFSWAQGTDTILADEMGLGKTIQTIVFLYSLYK
EGHTKGPFLVSAPLSTIINWEREFQMWAPKFYVVTYTGDKDSRAIIRENEFSFEDNAIKG
GKKAFKMKREAQVKFHVLLTSYELITIDQAALGSIRWACLVVDEAHRLKNNQSKFFRVLN
GYKIDHKLLLTGTPLQNNLEELFHLLNFLTPERFNNLEGFLEEFADISKEDQIKKLHDLL
GPHMLRRLKADVFKNMPAKTELIVRVELSPMQKKYYKYILTRNFEALNSRGGGNQVSLLN
IMMDLKKCCNHPYLFPVAAMESPKLPSGAYEGGALIKSSGKLMLLQKMLRKLKEQGHRVL
IFSQMTKMLDLLEDFLDYEGYKYERIDGGITGALRQEAIDRFNAPGAQQFCFLLSTRAGG
LGINLATADTVIIFDSDWNPHNDIQAFSRAHRIGQANKVMIYRFVTRASVEERITQVAKR
KMMLTHLVVRPGLGSKAGSMSKQELDDILKFGTEELFKDENEGENKEEDSSVIHYDNEAI
ARLLDRNQDATEDTDVQNMNEYLSSFKVAQYVVREEDKIEEIEREIIKQEENVDPDYWEK
LLRHHYEQQQEDLARNLGKGKRVRKQVNYNDAAQEDQDNQSEYSVGSEEEDEDFDERPEG
RRQSKRQLRNEKDKPLPPLLARVGGNIEVLGFNTRQRKAFLNAVMRWGMPPQDAFTTQWL
VRDLRGKTEKEFKAYVSLFMRHLCEPGADGSETFADGVPREGLSRQQVLTRIGVMSLVKK
KVQEFEHINGRWSMPELMPDPSADSKRSSRASSPTKTSPTTPEASATNSPCTSKPATPAP
SEKGEGIRTPLEKEEAENQEEKPEKNSRIGEKMETEADAPSPAPSLGERLEPRKIPLEDE
VPGVPGEMEPEPGYRGDREKSATESTPGERGEEKPLDGQEHRERPEGETGDLGKREDVKG
DRELRPGPRDEPRSNGRREEKTEKPRFMFNIADGGFTELHTLWQNEERAAISSGKLNEIW
HRRHDYWLLAGIVLHGYARWQDIQNDAQFAIINEPFKTEANKGNFLEMKNKFLARRFKLL
EQALVIEEQLRRAAYLNLSQEPAHPAMALHARFAEAECLAESHQHLSKESLAGNKPANAV
LHKVLNQLEELLSDMKADVTRLPATLSRIPPIAARLQMSERSILSRLASKGTEPHPTPAY
PPGPYATPPGYGAAFSAAPVGALAAAGANYSQMPAGSFITAATNGPPVLVKKEKEMVGAL
VSDGLDRKEPRAGEVICIDD

Mutation numbering corresponds with transcript variant 1, NM_001005273.

As no experimentally solved 3D-structure of CHD3 exists, we performed homology modeling using the modeling option with standard parameters in the YASARA[3] & WHAT IF[4] twinset. Several models of the ATPase/helicase domain were created. The best scoring model was based on template PDB-file 5JXR (M. thermophilia MtISWI, sequence identity 41% over the aligned residues), which shows a

closed conformation of the ATPase/helicase domain. We also studied the model based on PDB-file 3MWY (yeast Chd1, sequence identity 45%) which shows a more open conformation but contains an ATP substitute. Both templates represent an auto-inhibited form, however, it is impossible to say which of these models best represents the real biological form of CHD3, since movement of the domains is probably important for correct functioning of the protein. Therefore, both models were studied.

Results

Introduction of models and mutations

The complete CHD3 protein (2000 amino acids in isoform 1) is even bigger than the modeled domains shown here. Model 1 (closed conformation, based on 5JXR) contains residues 500-1290, while model 2 (open conformation, based on 3MWY) contains residues 445-1413.

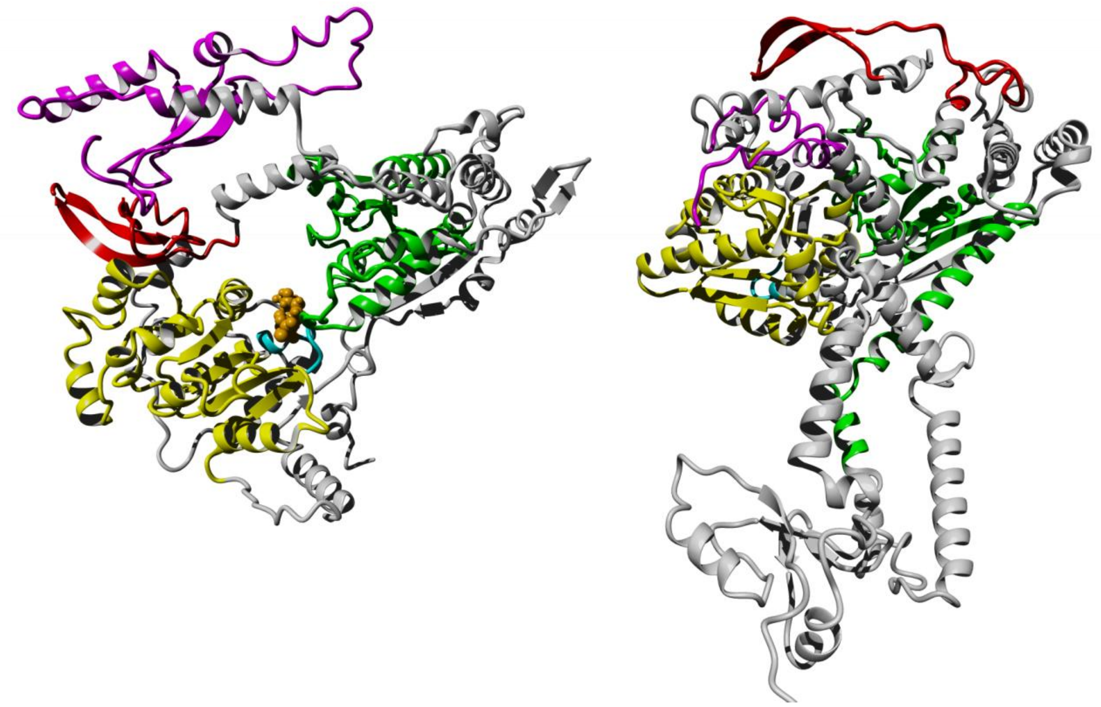

Fig 1: Overview of the two CHD3- models used in this study. Model 1 on the left represents the opened conformation with ATP (orange) bound. The model on the right (model 2) represents a more closed conformation, in which the two ATP-domains are interacting.

Domains indicated are: chromo domain 1 (494-595; magenta), chromo domain 2 (631-673; red), helicase ATP binding domain (yellow), helicase C-terminal domain (green), ATP binding residues (761-768; cyan). Grey residues do not belong to an indicated domain.

Figure 1 shows that the position of the domains relative to each other can possibly change depending on removal of inhibition and complex formation (with ATP, but probably also with other molecules,

co-factors and DNA). In the open conformation, a wide gap exists between the Helicase ATP-binding domain (yellow) and Helicase C-terminal domain (green), while in the closed conformation no gap is seen. The existence of at least two different conformations indicates that it is necessary to study the effects of the mutations in these two different models.

It is unclear what triggers the conformational change. The authors of the original articles that belong to the templates[5,6] speculate that the Chromodomains are important for auto-inhibition and differentiation between naked DNA and DNA with nucleosomes. If that is true, both models here represent an autoinhibited state. However, one of them contains ATP and the other one shows more interaction within the ATP binding domains.

Also, it might very well be possible that the closed conformation contains ATP while the open conformation does not (the opposite of what is shown here). This is one of the limitations of modeling; substrates present or missing in the template will also be present or missing in the model.

The missense mutations were mapped on the models as is shown in figure 2.

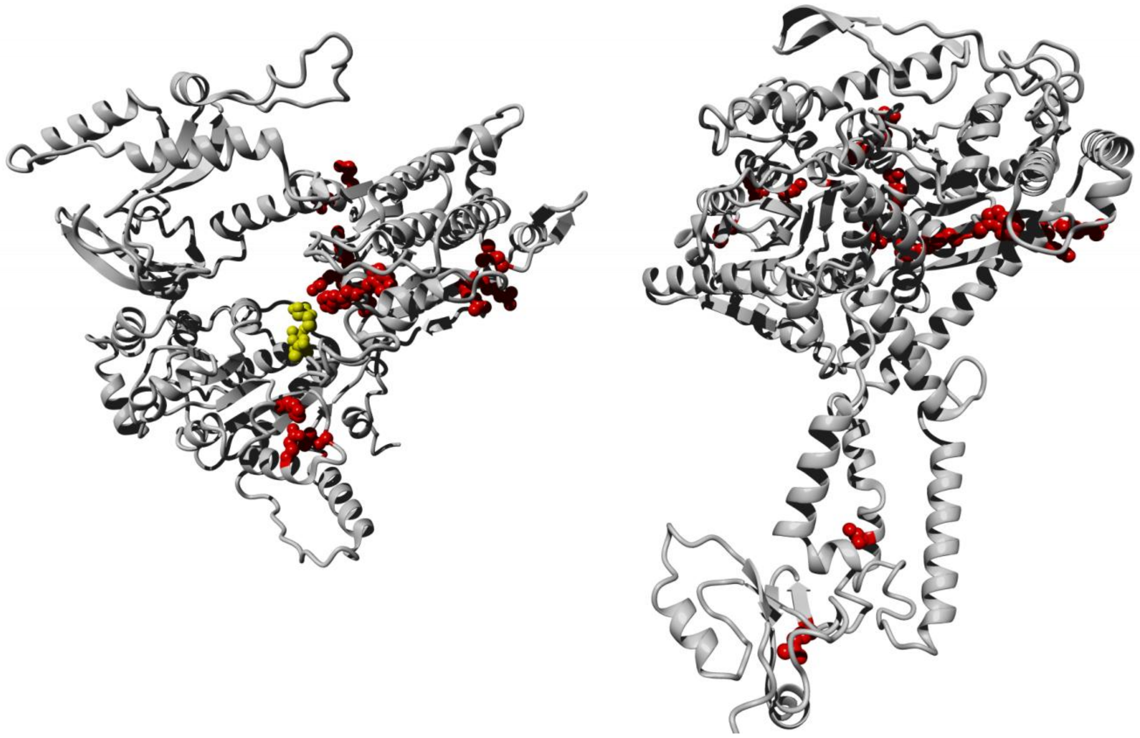

Figure 2 shows the position of all missense mutations studied here in both conformations. The positions of the mutated residues are indicated in red, the sidechains of these residues are shown as red balls. The ATP molecule is shown in yellow.

We performed a detailed analysis of the three-dimensional modeling of the mutations. As the CHD3 mutations are found largely clustered in the conserved motifs characteristic of the SF2 superfamily of helicases/translocases, we combined the 3D modeling analysis with information from the literature on these conserved motifs. A summary of this analysis is provided below.

His886Arg, Leu915Phe, Glu921Lys and Gly961Glu

	**Model 1 (open state, with ATP)**	**Model 2 (closed confirmation)**	**Conserved motifs?**
**His886Arg**	Located on interaction surface of Helicase ATP-binding domain. Might be responsible for correct interactions to facilitate ATP-binding. Mutation into an Arginine, which is bigger and positively charged, might affect the function of the protein domain.	Residue also located on surface, but does not interact with other half of Helicase domain. However, still in ATP-binding domain, and responsible for stabilizing interactions, which might be lost due to the mutation.	His886 is part of the Walker B motif in **motif II**. This motif coordinates the catalytic Mg++ involved in ATP hydrolysis.
**Leu915Phe**	Leu915 residue is semiburied, and in close contact with His886 and Glu921. It seems to make hydrophobic interactions that stabilize ATP-binding domain. A bigger residue like Phe will not fit and destabilizes the protein.	Leu915 is close to Glu921 and Gly961 (in this model His886 is a bit further away). Leu915 makes still hydrophobic interactions, probably important for interaction surface with other helicase domain. A Phe residue will not fit here without causing reorganisation of surrounding residues.	Leu915 is located in conserved **motif III**.
**Glu921Lys**	This residue is surrounded by His886, Gly961 and Leu915. It can be found at the surface but makes interactions that will be lost when mutated into Lys with opposite charge.	This residue is semiburied and makes hydrogenbonds and saltbridges, thereby stabilizing the domain. Close to Leu915 and His886. Mutation into Lys will destabilize the area since Lys carries an opposite charge and has a different shape.	Glu921 is located just next to **motif III**.
**Gly961Glu**	This residue clusters with His886, Leu915 and Glu921; although these other three are closer together. Gly is small and flexible, and located close to a Proline at the end of a helix. Mutation into Glu will introduce a much bigger and less flexible residue, this will cause a structural change that might affect the interaction surface and protein function.	Gly961 is still close to Leu915. It is also more clearly located on the surface that interacts with the other half of the helicase domain. Mutation might affect the local structure.	Gly961 is part of conserved **motif IV.**

Conclusion

These four residues are the only four mutated residues in the Helicase ATP-binding domain. In model 1 it is clearly visible that these four residues cluster together and mutation of one of them could affect the position of the others. Clustering is not so clear in the closed model (model 2), but the residues still appear in the same area. The two Helicase lobes are closer together in model 1, and therefore a close interaction with other mutated residues becomes visible as well. For example, in this model Asp1120 and Arg1121 are close to Gly961 and Leu915. Figure 3 and 4 below illustrate this clustering.

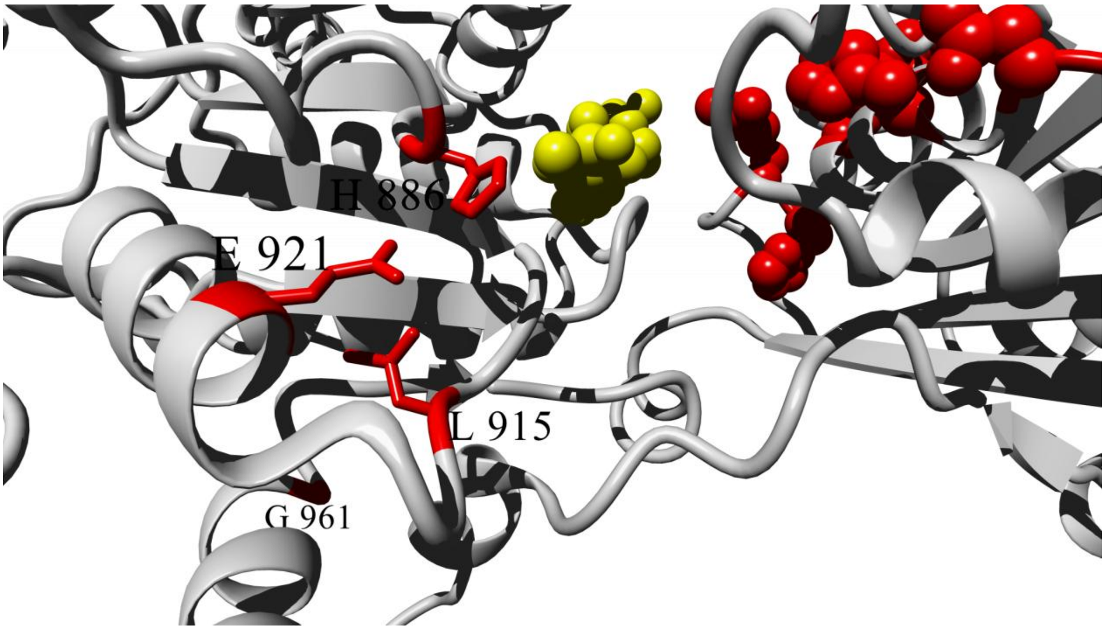

Figure 3: ATP bound model (model 1) based on 3MWY. In this model, residue H886, E921, L915 and G961 appear close together and might be responsible for the correct structure to bind ATP and to interact with the other half of the ATP binding domain (shown on the right with a subset of the other mutations shown as red balls) It seems possible that mutation of any of these residues would affect the correct conformation of this domain, which might affect ATP binding and/or correct interaction with the other half.

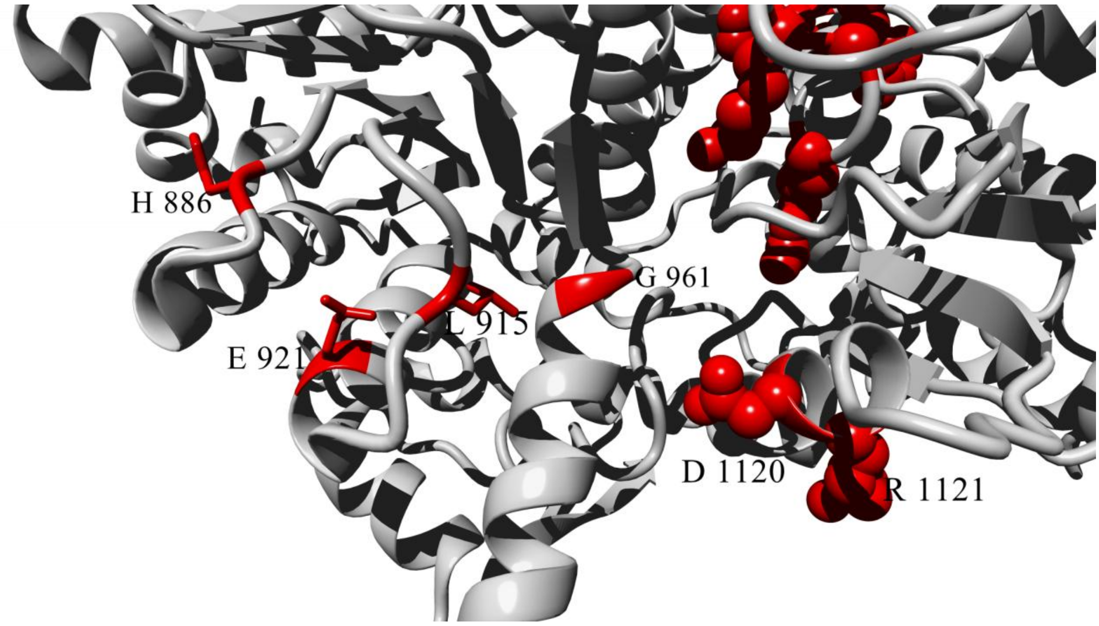

Figure 4: The ATP-free model (model 2) based on 5JXR shows the position of the residues mentioned above. Clustering in this model seems less obvious, but the residues are still located in the same domain. In this closed conformation, without ATP, it becomes clear how closely the mutations located in the two different helicase lobes could be located to each other (see the position of the labeled D1120 and R1121, other mutations in the other domain are shown as red balls).

Arg985Trp, Arg985Gln, Arg1187Pro and Leu1236Pro

	**Model 1 (open state, with ATP)**	**Model 2 (closed confirmation)**	**Conserved motifs?**
**Arg985Trp / Arg985Gln**	Arg985 is located on the surface of the Helicase C-terminal domain. It is not closely located to the ATP-binding or interaction surface. It makes a saltbridge and could be responsible for overall stability of this part of the protein. It clusters with Arg1187. Mutation of Arg into Trp might cause folding problems. Mutation into Glu would be easier, but stabilizing saltbridges would still be lost.	In this model the last C-terminal tail has a completely different conformation, which might be caused by the crystallization process. Originally, this model is a dimer with the last C-terminal tail swapped, and as a result Leu1236 is not close to Arg985 and Arg1187. Also, this model does not contain the C-terminal bridge (see Leu1326Pro), and Arg only seems to add some stable interactions to the area, contributing to general stable protein folding. Mutation into either Gln or Trp will affect this folding.	This mutation is located outside the canonical helicase motifs.
**Arg1187Pro**	This residue clusters with Arg985 on the surface of the protein's C-terminal helicase domain. It might mediate interaction with a regulatory unit. The residue makes a saltbridge, which will be lost due to the mutation. Mutation into Pro will change the backbone conformation. Regardless of interactions with a regulatory unit, this mutation will change local protein structure and might affect stability and function.	This residue clusters with Arg985 on the surface of the protein's C-terminal helicase domain. Mutation into Pro will change the backbone conformation, and will change local protein structure and might affect stability and function.	Arg1187 is part of **motif VI**, a motif that contains multiple mutations, and contributes to ATP binding and hydrolysis.
**Leu1236Pro**	In the ATP bound model, the residue is located closely to Arg985 and Arg1187 in a C-terminal bridge that is suggested to be important for regulation[5].	Leu1236 is found in a helix that is used for domain swapping in the crystal. The biological function of this helix is unclear. However, it is known that any mutation into Pro will affect the local backbone structure, because Pro is the only amino acid that will force the backbone into a rigid turn. Regardless of the exact position of the residue, this mutation can affect the protein's structure.	This mutation is located outside the canonical helicase motifs.

Conclusion

Residues Arg985, Arg1187 and maybe Leu1236 are found close together. It is unclear what the function of the last C-terminal tail is, although it has been suggested to have a regulatory effect[5]. In that case, interactions with the tail are important. If this is not the case, both mutations Arg1187Pro and Arg985Trp/Gln are expected to affect the structure and thereby maybe affect the function as well.

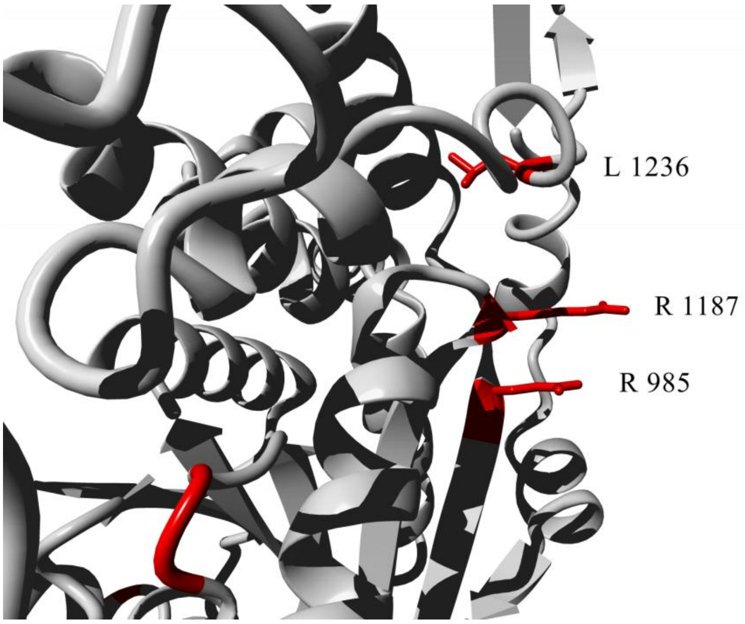

Figure 5: Positions of residue R985, R1187 and L1236 in the ATP-bound model. The three residues appear close together, although L1236 is officially not part of the helicase C-terminal domain.

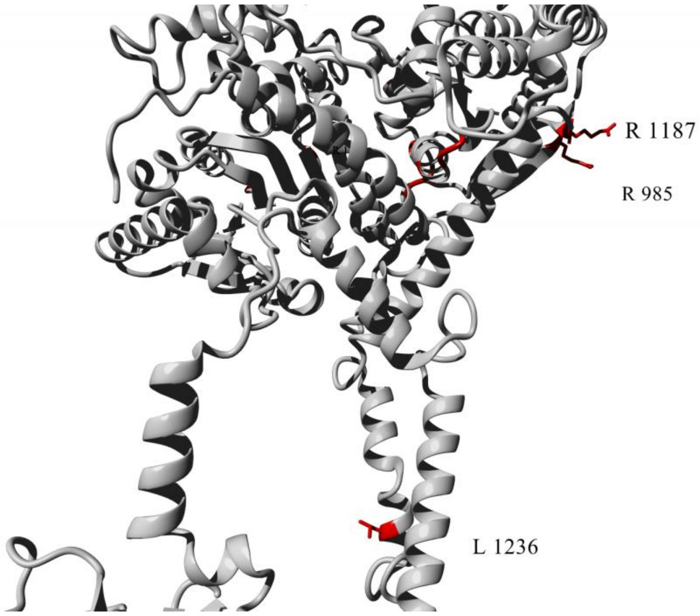

Figure 6: Positions of the residues R985, R1187 and L1236 in the ATP-free model. In this model, we can see that the L1236 residue is located in a very different position. This is due to the different position of the last C-terminal tail. It is unclear whether this tail has a biologically relevant function.

Asp1120His and Arg1121Pro

	Model 1 (open state, with ATP)	Model 2 (closed confirmation)	Conserved motifs?
**Asp1120His**	Asp1120 is part of a helix in the C-terminal helicase domain, and clusters with Arg1121. The residues are not especially close to the ATP, although Asp1120 seems to be located on the possible interaction surface. Asp is negatively charged, which is needed to make correct interactions, mutation into His will affect these interactions. Mutation might affect interaction between domains and/or ATP binding.	In this model Asp1120 gets close to the other half of the helicase domain (Helicase ATP-binding domain), for example close to mutated residue Gly961. Asp is negatively charged, which is needed to make correct interactions, mutation into His will affect these interactions. Mutation might affect interaction between domains and/or ATP binding.	Asp1120 is part of helix integral to conserved **motif V.** Mutations in Motif V in the context of yeast SNF2 abrogate ATP hydrolysis and remodeling activity.[7,8]
**Arg1121Pro**	This residue is located in the same helix in the C-terminal helicase domain as Asp1120, although its sidechain points in a different direction. The Arg sidechain is positively charged and makes hydrogen bonds and saltbridges. The Arg 1121 residue is probably not involved in ATP binding or interaction with the other domains, but the interactions it makes might be important for a stable structure. Mutation into Pro will surely affect the structure because interactions will be lost, and Pro will change the backbone conformation.	This residue is located in the same helix in the C-terminal helicase domain as Asp1120, although its sidechain points in a different direction. The Arg sidechain is positively charged and makes hydrogen bonds and saltbridges. The Arg 1121 residue is probably not involved in ATP binding or interaction with the other domains, but the interactions it makes might be important for a stable structure. Mutation into Pro will surely affect the structure because interactions will be lost, and Pro will change the backbone conformation.	Arg1121 is part of helix integral to conserved **motif V.** Mutations in Motif V in the context of yeast SNF2 abrogate ATP hydrolysis and remodeling activity.[7,8]

<u>Conclusion</u>

Asp1120 and Arg1121 are part of a helix in the C-terminal helicase domain, integral to conserved motif V. The mutations are expected to affect interaction between domains and/or ATP-binding (Asp1120His), and to affect the stable structure (Arg1121Pro). Mutations in Motif V in the context of yeast SNF2 affect ATP hydrolysis and remodeling activity.

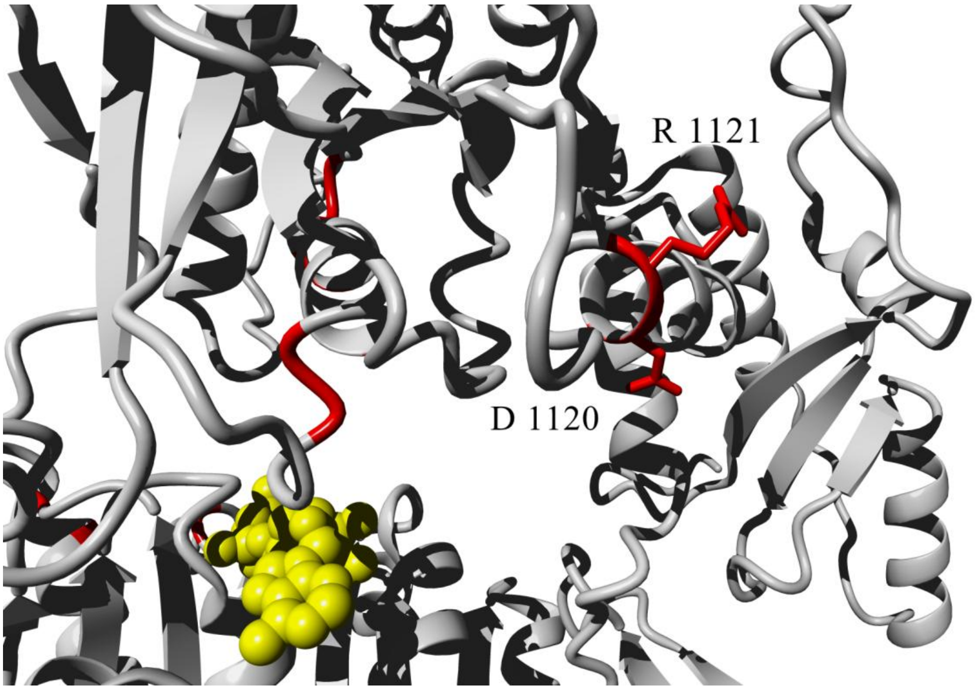

Figure 7: Positions of R1121 and D1120 in the ATP bound model (model 1). In this model, the residues are not located closely to the ATP, but could contribute to the interaction surface.

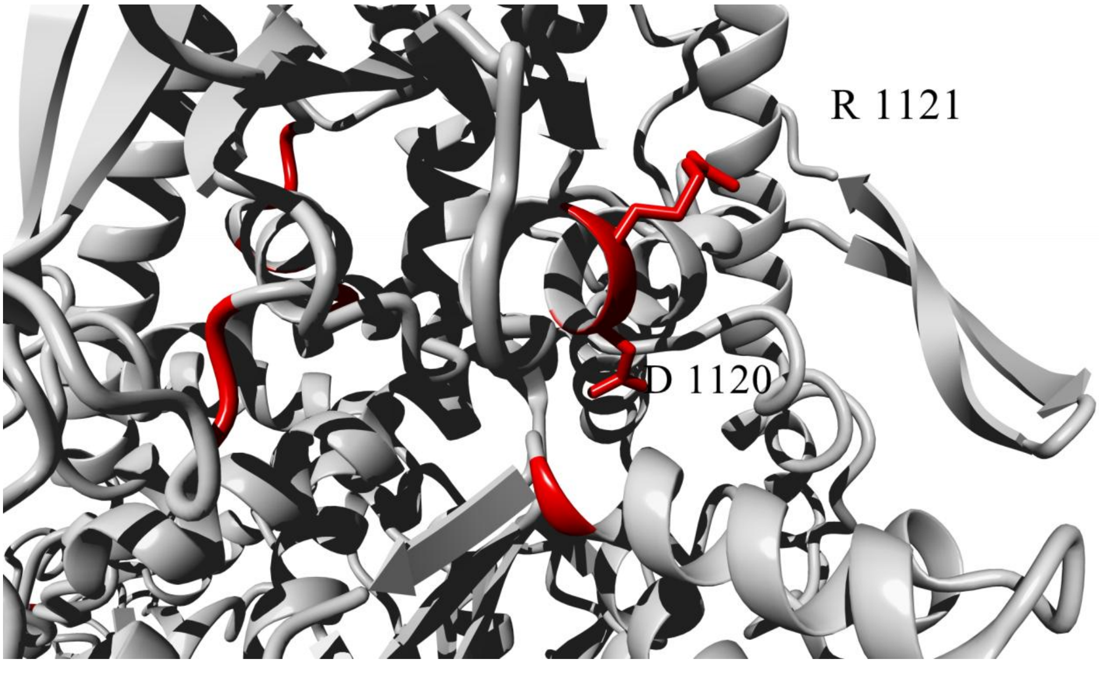

Figure 8: ATP-free model (model 2), shows the positions of D1120 and R1121. In this case we can see the close proximity of D1120 and G961 (the red residue in the helix right below D1120).

Thr1136Ile, Trp1158Arg, Asn1159Lys, His1161Arg

	**Model 1 (open state, with ATP)**	**Model 2 (closed confirmation)**	**Conserved motifs?**
**Thr1136Ile**	This residue is located in the core of the protein, making a hydrogenbond with its –OH group and hydrophobic interactions with the methyl in its sidechain. The tight packing does not allow a bigger residue here. Also, the mutation will cause loss of the hydrogenbond and thereby destabilize the local structure. The surrounding residues seem important for correct shape of the interaction site.	This residue is located in the core of the protein, making a hydrogenbond with its –OH group and hydrophobic interactions with the methyl in its sidechain. The tight packing does not allow a bigger residue here. Also, the mutation will cause loss of the hydrogenbond and thereby destabilize the local structure. The surrounding residues seem important for correct shape of the interaction site.	This amino acid is located within **Motif V**. Mutations in Motif V in the context of yeast SNF2 abrogate ATP hydrolysis and remodeling activity.
**Trp1158Arg**	This residue is clearly important for the hydrophobic core of the protein. In both models it is (semi) buried and makes hydrophobic interactions. Mutation into anything else will affect the stability and the structure of this domain.	This residue is clearly important for the hydrophobic core of the protein. In both models it is (semi) buried and makes hydrophobic interactions. Mutation into anything else will affect the stability and the structure of this domain.	This Trp residue has recently been shown to be of critical importance in remodeling, by binding nucleosomal DNA in the minor groove. Mutation of this residue impacts remodeling, not ATP hydrolysis[9,10].
**Asn1159Lys**	This residue is located in the same helix as some of the following mutations (see below) and seems to form the interaction surface with ATP and the other domain. It makes a few hydrogenbonds. Mutation into something larger and positively charged might affect interactions.	This residue is located in the same helix as some of the following mutations(see below). This residue is buried and makes many interactions, some of the interactions are made with residues in a N-terminal helix. This helix might be important for auto-inhibition[6]. Mutation into something larger and positively charged might affect interactions with surrounding residues or with the inhibition helix.	This amino acid is located adjacent to the Trp residue at position 1158, and might alter the environment of this critical Trp residue.
**His1161Arg**	This residue follows a similar story to the mutations above. It is located in the same helix as Asn1159 and seems to be important for the surface interactions. Mutation into Arg will change the amino acid properties drastically.	This residue follows a similar story to the mutations above. It is located in the same helix as Asn1159 and seems to be important for the surface interactions. In this model the residue makes interactions with the putative inhibition helix. Mutation into Arg will change the amino acid properties drastically.	This amino acid is located close to the Trp residue at position 1158, and might alter the environment of this critical Trp residue.

Conclusion

Based on recent literature, it is known that the Trp residue at position 1158 is of critical importance in remodeling. The Trp binds nucleosomal DNA in the minor groove, and mutation of this residue will impact chromatin remodeling. The other two residues (1159 and 1161) are probably altering the environment of this critical Trp residue.

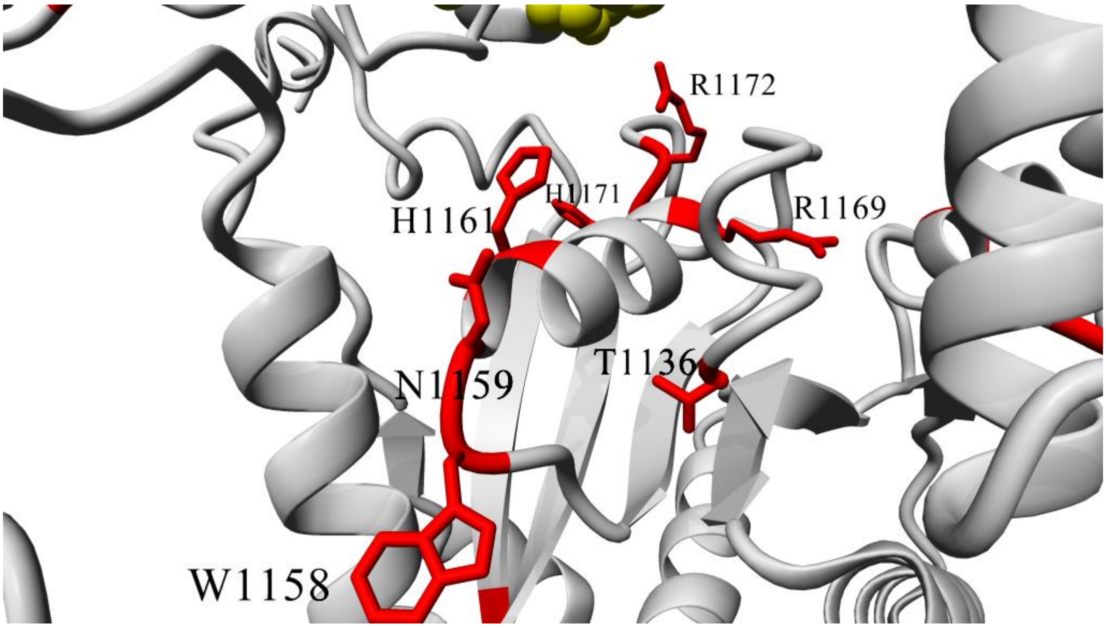

Fig 9: Overview of the remaining mutations in the core of the protein. Most of these mutations are in the ATP-bound open model located on the surface which becomes buried in the closed model. T1136 is located below this surface, but is required for correct positioning of the other residues.

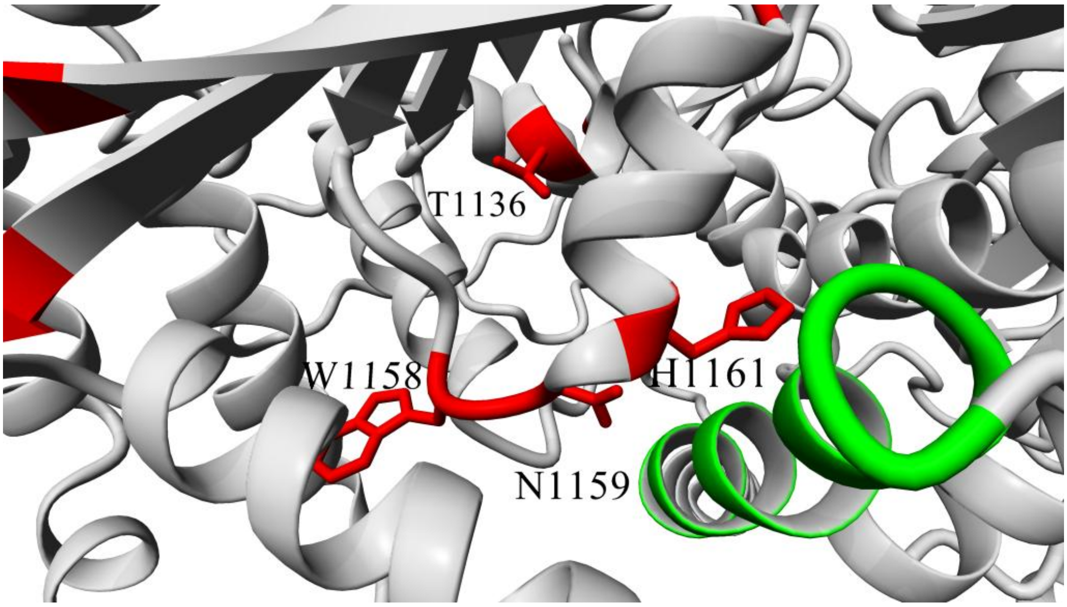

Fig 10: Mutations T1136,W1158, N1159 and H1161. The W1158 is buried and makes many hydrophobic interactions. N1159 and H1161 become buried in the closed model and seem to interact with residues in a putative inhibition helix (green)[6]. Recent articles show that W1158 is of critical importance in binding nucleosomal DNA[9,10].

Arg1169Trp, His1171Arg and Arg1172Gln

	**Model 1 (open state, with ATP)**	**Model 2 (closed confirmation)**	**Conserved motifs?**
**Arg1169Trp**	This residue is buried in both models. The residue might be important for correct shaping of the interaction surface.	This residue is buried in both models. In this model the residue is so buried that a bigger Trp will never fit.	This mutation is part of **Motif VI**. Arg1169 is an Arginine finger that is thought to be critical for ATP binding and catalysis.
**His1171Arg**	This residue is located close to Arg1172 and Arg1169, and also responsible for the correct interaction surface. Mutation of His to Arg introduces a bigger residue with positive charge. This will change the interaction surface.	In this model the residue becomes buried (see figure 11). Mutation of His to Arg introduces a bigger residue with positive charge. This residue will not fit and damages interactions made by His. This mutation will affect protein structure and function.	This mutation is part of **Motif VI**, a critical motif for ATP binding and catalysis.
**Arg1172Gln**	This mutation might have a similar effect as the mutations above. It is located on the possible interaction surface between the two halves of the helicase domain. Interactions of the Arg will be lost because Gln will not have the same size and charge.	This mutation becomes buried in the closed model. Interactions of the Arg will be lost because Gln will not have the same size and charge.	This mutation is part of **Motif VI**. Arg1172 is an Arginine finger that is thought to be critical for ATP binding and catalysis.

<u>Conclusion</u>

These three mutations are part of Motif VI, a motif that contributes Arginine fingers (Arg1169 and Arg1172) critical for ATP binding and catalysis. Based on three-dimensional modeling, these three mutations are important for correct shaping of the interaction surface.

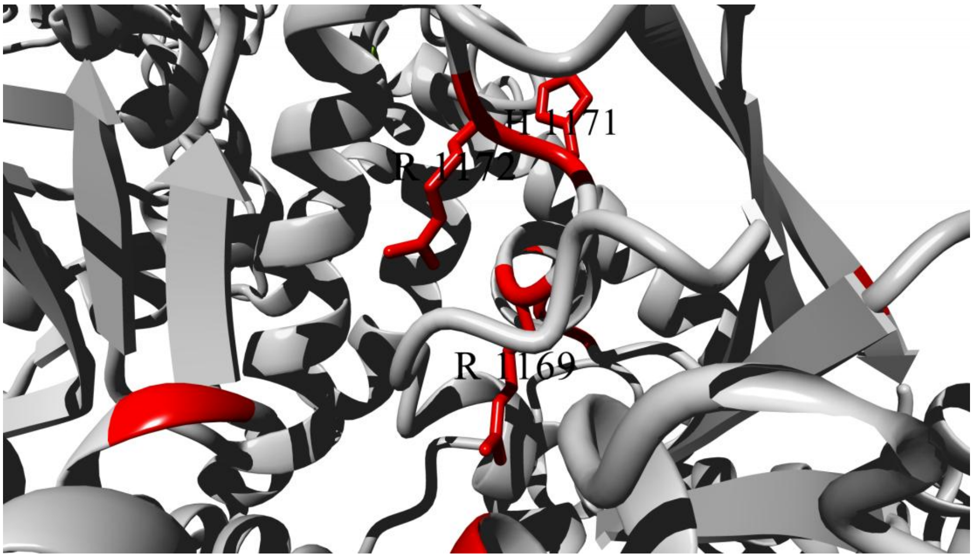

Fig 11: Residues R1169, H1171 and R1172 in the closed model show that these residues are buried and that any mutation here can damage the protein structure. See Figure 9 for the modeling of these three mutations in the open model (model 1).

Arg1342Gln and Arg1881Leu

Arg1342Gln

This residue is unfortunately not located in the modeled domain. No information or function is known for this residue or this region and therefore it is difficult to predict the effect of the mutation. Arg and Gln are both hydrophilic, but Arg is postively charged while Gln is neutral. Arg is also is bigger than Gln. Depending on the interactions made by the wild-type, this mutation can be damaging.

Arg1881Leu

This residue is not even close to the modeled helicase domains. Again, it is difficult to predict an effect without structural knowledge. However, the properties of Arg and Leu amino acids are very different. Arg is larger, positively charged and hydrophilic. Leu is smaller, neutral and hydrophobic. The differences in properties between these residues can strongly affect the protein structure.

Gly1109del

This mutation is the only deletion of 1 residue in patients known so far. It deletes a flexible Gly residue in a contact loop. The residue is not located on the interaction surface. Instead, it seems to be important for a particular loop structure that interacts with residues in helix 611-617. Gly itself does not have a sidechain, and therefore is the most flexible residue. The fact that residue 1108 is also a Glyc indicates that this loop might need more flexilbiity. The interaction with the 611-617 helix occurs in both models, although the helix seems much closer to the residue in the closed model.

The effect of this mutation is difficult to predict without knowing the exact function of the loop and the interacting helix. Deletion of 1 residue would result in a shorter loop, but it will not affect folding of the remaining protein. Interaction between the helix and the loop might be required for regulation, but more information is needed.

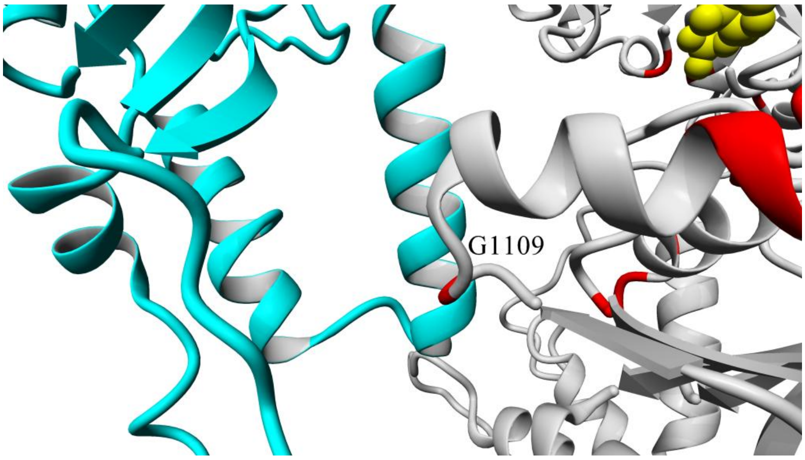

Fig 12: Position of G1109 in the open structure with ATP. G1109 and its surrounding residues interact with residues 611-617 in the helix (cyan). The function of this interaction is not known but will be affected by the mutation.

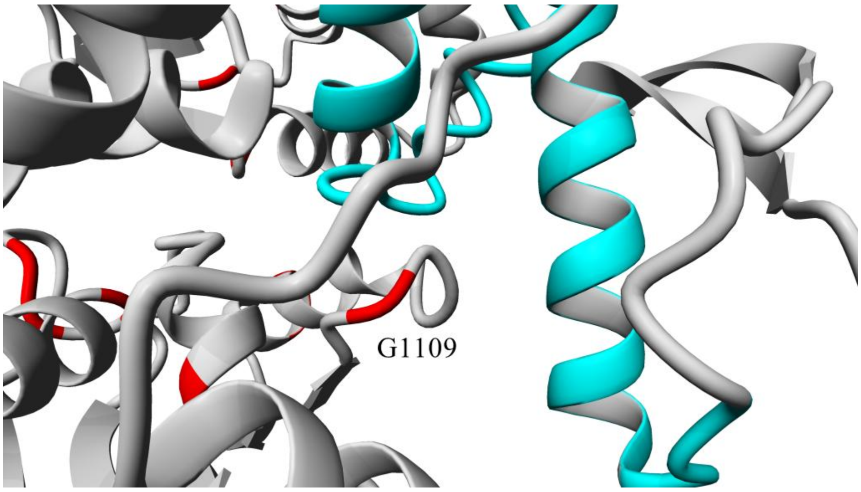

Fig 13: Position of G1109 in the closed model. The loop that contains 1109 is still close to the helix with residues 611-617. However, the position of the helix (and the domain that contains this helix) has changed and as a result the interaction is less close.

Supplementary References

1. Weiss, K. *et al.* De Novo Mutations in CHD4, an ATP-Dependent Chromatin Remodeler Gene, Cause an Intellectual Disability Syndrome with Distinctive Dysmorphisms. *Am J Hum Genet* **99**, 934-941 (2016).
2. Sifrim, A. *et al.* Distinct genetic architectures for syndromic and nonsyndromic congenital heart defects identified by exome sequencing. *Nat Genet* **48**, 1060-5 (2016).
3. Krieger, E., Koraimann, G. & Vriend, G. Increasing the precision of comparative models with YASARA NOVA--a self-parameterizing force field. *Proteins* **47**, 393-402 (2002).
4. Vriend, G. WHAT IF: a molecular modeling and drug design program. *J Mol Graph* **8**, 52-6, 29 (1990).
5. Hauk, G., McKnight, J.N., Nodelman, I.M. & Bowman, G.D. The chromodomains of the Chd1 chromatin remodeler regulate DNA access to the ATPase motor. *Mol Cell* **39**, 711-23 (2010).
6. Yan, L., Wang, L., Tian, Y., Xia, X. & Chen, Z. Structure and regulation of the chromatin remodeller ISWI. *Nature* **540**, 466-469 (2016).
7. Xia, X., Liu, X., Li, T., Fang, X. & Chen, Z. Structure of chromatin remodeler Swi2/Snf2 in the resting state. *Nat Struct Mol Biol* **23**, 722-9 (2016).
8. Smith, C.L. & Peterson, C.L. A conserved Swi2/Snf2 ATPase motif couples ATP hydrolysis to chromatin remodeling. *Mol Cell Biol* **25**, 5880-92 (2005).
9. Farnung, L., Vos, S.M., Wigge, C. & Cramer, P. Nucleosome-Chd1 structure and implications for chromatin remodelling. *Nature* (2017).
10. Liu, X., Li, M., Xia, X., Li, X. & Chen, Z. Mechanism of chromatin remodelling revealed by the Snf2-nucleosome structure. *Nature* **544**, 440-445 (2017).
