# Supplementary material for: CHD3 helicase domain mutations cause a neurodevelopmental syndrome with macrocephaly and impaired speech and language
Source: Nat Commun. 2018 Nov 5;9:4619. doi: 10.1038/s41467-018-06014-6 (PMC6218476; doi:10.1038/s41467-018-06014-6)
Supplement: Supplementary file 3 — Description of Additional Supplementary Files [file 41467_2018_6014_MOESM3_ESM.pdf]

Description of Additional Supplementary Files

File Name: Supplementary Data 1
Description: Clinical features and mutation details of individuals with de novo CHD3 mutations.

File Name: Supplementary Data 2
Description: Primary data of ATPase experiments.
